# Transcriptomics and network pharmacology reveal the molecular mechanisms through which leonurine protects against *Salmonella enteritidis* infection in IEC-6 cells

**DOI:** 10.3389/fmicb.2025.1665245

**Published:** 2026-04-10

**Authors:** Li Yang, Chenghui Xu, Jie Wei, Ruixue Sa, Yuanyuan Yuan, Lining Xia, Huijun Shi, Qiang Fu

**Affiliations:** 1 College of Veterinary Medicine, Xinjiang Agricultural University, Ürümqi, China; 2 Xinjiang Key Laboratory of Animal Disease Research, Institute of Veterinary Medicine, Xinjiang Academy of Animal Sciences, Ürümqi, China

**Keywords:** leonurine, *Salmonella enteritidis*, invasion, mechanism, transcriptomics, network pharmacology, IEC-6 cells

## Abstract

Leonurine has been demonstrated to exert significant therapeutic effects against *Salmonella enteritidis* induced intestinal inflammation; however, its precise mechanism of action in combating *Salmonella* infection remains unclear. In this study, a *Salmonella* infection IEC6 cell model was established to evaluate the efficacy of leonurine in combating *Salmonella* infection using CCK8, cytopathic effect (CPE) analysis, immunofluorescence, transmission electron microscopy (TEM), and flow cytometry. Subsequent integration of network pharmacology, transcriptomics, and molecular docking revealed potential targets and signaling pathways, which were further validated by RT–qPCR and Western blotting. Immunofluorescence and TEM revealed that leonurine effectively inhibited bacterial invasion and intracellular proliferation. Flow cytometry and CPE assays demonstrated its ability to alleviate cellular damage and inhibit apoptosis. The integrated network pharmacology transcriptomics molecular docking analysis revealed robust interactions between leonurine and PTGS2, PARP1, and mTOR. Mechanistic validation confirmed that leonurine inhibits *Salmonella* invasion via the modulation of tight junction proteins and restricts intracellular bacterial proliferation by regulating ferroptosis and autophagy signaling pathways. This study reveals the potential targets and molecular mechanisms through which leonurine combats *Salmonella* infection, providing a scientific foundation for the prevention and control of salmonellosis.

## 1 Introduction

Salmonella, a gram-negative pathogen, primarily infects livestock through contaminated food sources and can cause mild to severe illness ranging from diarrhea and vomiting to death. *Salmonella* infection causes significant economic losses to the livestock breeding industry. In clinical settings, 60% of *Salmonella* strains exhibit anti-biotic resistance (Murdoch et al., 1998). Antibiotic resistance is often associated with increased morbidity and mortality, as it leads to a delay in initiating appropriate treatment. In addition, antibiotics will become less effective as resistance increases. Therefore, new therapeutic agents are needed to combat *Salmonella* infection. Traditional Chinese medicine extracts are less toxic and have fewer side effects and may be able to prevent *Salmonella* infection.

The chemical composition of Chinese herbal medicines is completely different from that of antibiotics, which have become the main driver of drug resistance in bacteria. In recent years, Chinese herbal medicine has achieved some success in terms of combating bacterial infection (Liu et al., 2010). For example, the isoflavone berberine in *Coptis* coptidis and cyanidin in Isatidis roots have inhibitory effects on a variety of bacteria and viruses (Chen et al., 2021). The alkaloid leonurine extracted from motherwort has been shown to have significant antiinflammatory, antioxidation and antiapoptotic effects in basic research and clinical applications (Qiu et al., 2021). In recent studies, leonurine (Leo) was shown to significantly inhibit bacterial adhesion, resulting in antibacterial effects (Luo et al., 2020; Micota et al., 2014; Maifo, 2021). Elucidating how Leo acts on multiple targets to exert an inhibitory effect on bacterial infection has been particularly challenging. Network pharmacology combined with multiomics, such as transcriptomics, has been widely used to analyze the targets and molecular mechanisms of drugs in the treatment of diseases (Shi et al., 2022; Wang et al., 2024; Hu et al., 2023).

Our previous studies revealed that Leo can significantly decrease lipopolysaccharide (LPS) production in poultry with intestinal inflammation (Yang et al., 2019). Therefore, we developed a novel integrative strategy based on transcriptomics and network pharmacology to further elucidate the mechanism by which Leo affects *Salmonella enteritidis* infection. We initially analyzed its regulatory network and core targets via network pharmacology and molecular docking. We subsequently combined the results of the transcriptomic analysis and cellular experiments to reveal and verify the mechanisms by which Leo combats *Salmonella* infection. In this study, the potential targets and mechanisms through which Leo protects against *Salmonella* infection were revealed via a combination approach, providing theoretical and data support for its further development and clinical application.

## 2 Materials and methods

### 2.1 Strain identification and IEC-6 cell culture and infection

Intestinal epithelial cell-6 cells (Conservation Genetics CAS Kunming Cell Bank, China, KCB200720YJ) were cultured in a 10 cm^2^ dish with complete medium (89% DMEM, 10% fetal bovine serum, and 1% penicillin–streptomycin solution) until they reached 90% confluence. The cells were then digested with trypsin, passaged, and incubated at 37 °C with 5% CO_2_ in a carbon dioxide incubator during the logarithmic growth phase for good cell testing. *Salmonella enteritidis* was obtained from the College of Veterinary Medicine, Xinjiang Agricultural University, China. The strains were inoculated into Meuller–Hinton (MH) medium and placed in a constant-temperature shaking incubator at 37 °C and 180 rpm overnight three times for sequencing of the 16S rRNA gene region. The returned sequences were analyzed using the NCBI blast ratio^1^ by downloading article #20 of the nucleic acid sequence followed by MEGA7 software analysis to construct a genetic evolutionary tree. The experimental groups included a blank group, a Leo group, a *Salmonella* group, a Leo + *Salmonella* treatment group and a *Salmonella* alone treatment group. The multiplicity of infection (MOI) was = 100:1, and IEC-6 cells were cultured in DMEM supplemented with 2% FBS.

### 2.2 CCK-8 assays

Leo obtained from the National Institutes for Food and Drug Contral, China, (111823, 111823-202206) was used, the content was calculated as 98.1% and stored in Dimethyl sulfoxide (DMSO). The cells were transferred to a 96-well cell culture plate, and 100 μl of cell suspension was added to each well. Each group was set up with three duplicate wells and cultured overnight. The cells were subsequently cultured for 2, 4, 6, 12, or 24 h with Leo solution at final mass concentrations of 5, 10, 20, 40, or 80 μmol/L, respectively. After completion of the coculture period, the culture medium in the wells of the cell culture plate was replaced with fresh medium, followed by the addition of CCK-8 solution (10 μl per well) included in the CCK-8 kit (BioDEE Biotechnology Co., Ltd., Beijing, China). The plates were then incubated in a cell incubator for 2 h. Following incubation, cell viability was determined by measuring the absorbance at a wavelength of 450 nm with a microplate reader and accurately recording the optical density (OD) values.

### 2.3 Adhesion, invasion, and proliferation assays

To study the effect of Leo on *S. enteritidis* invasion of host cells, MH medium was used for *S. enteritidis* infection at an MOI = 1:100. The cells were inoculated in 2% medium (98% DMEM and 2% fetal bovine serum). One percent of the cells were then inoculated with bacteria and cocultured with Leo at final molar concentrations of 40, 60, and 80 μmol/L. Three experimental set ups were established to evaluate the adhesion, invasion, and proliferation of *S. enteritidis*, and the control, administration, challenge, and treatment cell groups were established accordingly.

In the adhesion experiment, the bacterial mixture was centrifuged and resuspended in 2% medium at a final concentration of 1 × 10^7^ CFU/mL. Two milliliters of the bacterial mixture was added to each well of a 6-well plate, followed by Leo. The cells were cultured at 37 °C with 5% CO_2_ for 2 h for bacterial adherence. After another 2 h, the cells were washed 5 times with preheated PBS to remove nonadherent bacteria. Trypsin was added and incubated for 20 min to lyse the cells and release bacteria. The suspension was diluted and plated on SS agar plates and incubated over-night. The adhesion rate was calculated using the following formula: (number of adhering bacteria/initial total number of bacteria) × 100%.

In the invasion experiment, after 2 h of bacterial infection, the cells were washed 5 times with preheated PBS and then incubated with 2% medium supplemented with 100 μg/mL gentamicin for 1 h to clear the extracellular bacteria. The remaining steps were the same as those for adhesion. The invasion rate was calculated using the following formula: (number of invading bacteria/initial total number of bacteria) × 100%.

In the proliferation experiment, after incubation in medium supplemented with 2% or 100 μg/mL gentamicin as performed in the invasion experiment, the cells were washed with PBS and cultured for 23 h in medium supplemented with 2% or 10 μg/mL gentamicin. The subsequent steps were the same as those for the adhesion experiment. The proliferation rate was calculated using the following formula: (number of proliferating bacteria/initial total number of bacteria) × 100%.

### 2.4 Immunofluorescence assay

The staining for *S. enteritidis* is carried out using the FITC-L-Dys staining kit (I0201, Beyotime Biotechnology, Shanghai, China). Dilute the 1 mM stock solution with PBS to 50 nM. Shake the bacteria overnight. Discard the culture medium, wash with PBS once. Add the working solution of FITC-L-Dys (50 nM), incubate at 37 °C in the dark for 60 min, and wash with PBS once. Coverslips were added to each well of a 24-well plates, and the cells were then added to the wells. When the cells reached 80% confluence, the culture medium was discarded, and the control group, 60 μmol/L Leo treatment group, *S. enteritidis* challenge group, and 60 μmol/L Leo treatment group were established. The cells in the blank group were cultured in 2% medium (98% DMEM, 2% fetal bovine serum), the cells in the 60 μmol/L Leo treatment group were cultured in 2% medium supplemented with 60 μmol/L Leo, the cells in the *S. enteritidis* challenge group and 60 μmol/L Leo treatment group were cultured in 2% medium supplemented with *S. enteritidis* (MOI of 1:100), and the 60 μmol/L Leo treatment groups were cultured in medium supplemented with 60 μmol/L Leo. The cells were treated for 2, 4, 6, 8, or 10 h, respectively. After sample collection, the liquid in the 24-well plate was discarded, and the samples were placed on a horizontal shaker and washed with PBS for 5 min each time. After the washing solution was discarded, 4% paraformaldehyde was added, and the samples were fixed at room temperature for 30 min. Then, three PBS washes were per-formed. Permeabilization was performed by adding 200 μl of 0.5% Triton X-100 for 10 min, 4% paraformaldehyde, Triton X-100, DAPI staining solution, and CoraLite594-conjugated goat anti-rabbit IgG (H + L) (BioDEE Biotechnology Co., Ltd., Beijing, China). Three additional washes with PBS were performed, followed by the addition of 200 μL of blocking buffer for 1 h. This was followed by three PBS washes. Finally, the coverslips were inverted onto glass slides coated with specific antibodies and incubated overnight at 4 °C. The next day, the coverslips were removed and washed three times with PBS for 10 min each on the horizontal shaker, and the speed of the shaker was less than 100 r/min. The secondary antibody was added, and the cells were incubated in the dark for 1 h at room temperature on a horizontal shaker. The cells were subsequently washed 3 times with PBS, 200 μL of DAPI staining solution was added, the cells were stained for 20 min, washed 3 times with PBS, sealed, and observed via confocal microscopy.

### 2.5 Transmission electron microscopy

Transmission electron microscopy was used to observe whether *S. enteritidis* invaded IEC-6 cells at 6 h of challenge and whether Leo could inhibit the proliferation of *S. enteritidis*. Two groups were set up: the *S. enteritidis* group and the Leo + *S. enteritidis* treatment group. After the model was successfully established, the cells were washed twice with PBS, digested with trypsin, placed in electron microscope fixation solution, and stored at 4 °C. The cells were subjected to lead and uranium double staining. The corresponding copper mesh of the cells was prepared via negative staining using the standard operating procedure and other experiments, photographed using a transmission electron microscope (HITACHI HT770, Japan), and observed using a Hitachi transmission electron microscope imaging system.

### 2.6 Cytopathic effect analysis

The control group, 60 μmol/L Leo treatment group, *S. enteritidis* challenge group, and 60 μmol/L Leo treatment group were established, and a Senescence β-Galactosidase Staining Kit (C0602, Beyotime Biotechnology, Shanghai, China) was used. The cells in the four groups were pretreated at an MOI of 1:100. *Salmonella enterica* was inoculated in 2% medium (98% DMEM, 2% fetal bovine serum) and added to 6-well plates to culture the cells 2, 4, 6, 8, or 10 h. After treatment, the culture medium was removed, the cells were gently washed once with PBS, and the cells were treated with reagents from the Senescence β-Galactosidase Staining Kit. One milliliter of β-galactosidase staining fixator was added and then fixed at room temperature for 15 min, after which the cell fixator was removed, and the cells were washed three times with PSS for 3 min each. After the removal of the PBS, 1 ml of the staining working solution was added to each well. Six-well plates were sealed with a sealing membrane to prevent evaporation, incubated overnight at 37 °C, and examined under an inverted microscope.

### 2.7 Flow cytometry

The control group, 60 μmol/L Leo treatment group, *S. enteritidis* challenge group, and 60 μmol/L Leo treatment group were established. The cells in the four groups were inoculated with *Salmonella* in 2% medium (98% DMEM, 2% fetal bovine serum) at an MOI of 1:100 6 h, and apoptosis induced by Leo inhibition of *S. enteritidis* was evaluated using an Annexin V-FITC Apoptosis Detection Kit and flow cytometry (C1062M; Beyotime Biotechnology, Shanghai, China). Dilute 4 × binding buffer to 1 × buffer with PBS, remove the remaining PBS from the centrifuge tube, add 100 μL of 1 × binding buffer to each tube, blow the cells with a pipetting gun to fully resuspend the cells, and add the dye under dark condition. The single staining group was added with Annexin V or PI 5 μL, and the Annexin V and PI double staining groups were added with Annexin V and PI 5 μL each, and gently mixed with a pipetting pistol. Annexin V-FITC/PI apoptosis detection kit uses FITC-labeled Annexin V as a probe. The maximum excitation wavelength of FITC was 488 nm, and the maximum emission wavelength was 525 nm. The green fluorescence of FITC was detected in FL1 channel. The PI-DNA complex had a maximum excitation wavelength of 535 nm and a maximum emission wavelength of 615 nm, and the red fluorescence of PI was detected in FL2 or FL3 channels. By software analysis, two-dispersion plots were drawn, with FITC on the abscissa and PI on the ordinate.

### 2.8 Transcriptome sequencing and real-time fluorescent quantitative PCR

Each group had three replicate groups. The control group, the 60 μmol/L Leo treatment group, the *S. enteritidis* challenge group, the 60 μmol/L Leo treatment group, and the *S. enteritidis* treatment group were inoculated with *Salmonella* in 2% medium (98% DMEM, 2% fetal bovine serum) at an MOI of 1:100 6 h. Total RNA was extracted. After quality inspection by Agilent 2100 (RIN ≥ 8.0), mRNA was enriched by oligo (dT) magnetic beads and a strain-specific library was constructed. 150 bp double-ended sequencing was performed on the Illumina NovaSeq 6000 platform. The original FASTQ data were evaluated for quality by FastQC and low-quality sequences (Phred < 20) were filtered by Trimmomatic. The reads after quality control were aligned to the reference genome using HISAT2, and the gene expression was quantified through feature Counts annotation. Differential expression analysis was conducted using DESeq2 (with log2FC > 1 and padj < 0.05 as the significance criteria), and ultimately GO/KEGG functional enrichment was performed through cluster Profiler (p.adjust < 0.05). Total RNA was extracted using TRIzol reagent, and the concentration and purity of the RNA were measured using a nucleic acid quantification instrument. The primer sequences are shown in Table 1. For the fluorescent quantitative PCR system, the total volume was 20 μL, and the reagents and concentrations are shown in Table 2, The PCR reaction program is set up in accordance with the instructions of the kit.

**TABLE 1:** Primer sequence.

Gene	Sequence	Amplified fragment size/bp
PTGS2	F: 5′-TCATAAGCGAGGACCTGGGTTC-3′	166
	R: 5′-GTCTTTGACTGTGGGGGGATAC-3′	
mTOR	F: 5′-AATCCCTCTCTCACCACCACAC-3′	193
	R: 5′-TGGCTCTTCACAAAGGACACC-3′	
PARP1	F: 5′-AAGGCAGCAGTGAATCCCA-3′	121
	R: 5′-ACCTTGGCCTGCACGCTGT-3′	
BPI	F: 5′-AAGGGTCTGGACTTCGTGTG-3′	180
	R: 5′-TAGCAGCTTGATCTGGGGAT-3′	
Tnf	F: 5′-CTGAACTTCGGGGTGATCG-3′	157
	R: 5′-CCTCTGCTTGGTGGTTTGC-3′	
Lcn2	F: 5′-GCTACGACGTGCAAGTGG-3′	200
	R: 5′-GGGGACAGAGAAAACGATGTT-3′	
THBS1	F: 5′-GGAACCTCCCAAAATGACCCT-3′	189
	R: 5′-GGACTGGTAGCCGAAAACAAA-3′	
HSD11B1	F: 5′-GGTGCTCTGCCTGGGTTACTA-3′	109
	R: 5′-TTCTCTTCCGATCCCTTTGCT-3′	
IER3	F: 5′-AGATTTTCACCTTCGACCCCC-3′	219
	R: 5′-GCGACACACCTTCTTCAGCCA-3′	
CCL7	F: 5′-AATTCATCCACTTGCTGCTAT-3′	84
	R: 5′-CCGACTACTGGTGATCTTTC-3′	
Sesn3	F: 5′-ACTTTCCACTCGTTTCCTCAC-3′	129
	R: 5′-TGGCAAATGTTCTTCTCCTCG-3′	
CP	F: 5′-GCAGATGGGAGACAGAAAGA-3′	246
	R: 5′-GCTGAACAAATACCACACGA-3′	
AREG	F: 5′-CCATAATTGTCTTTGTCTCCG-3′	101
	R: 5′-TTCTGCTTCTTCATATTCCCT-3′	
EREG	F: 5′-TGATTCTCGTTTTCCTCTTTCTT-3′	101
	R: 5′-GTATTCTTCCCTTGATTTTTTAC-3′	
ENPP2	F: 5′-TCAACGTAATAAGTGGACCGAT-3′	191
	R: 5′-GAAGGAAGACACAGAGAGGGGA-3′	
BDKRB1	F: 5′-TCCCCACATTCCTTCTACG-3′	111
	R: 5′-TCAACTCCACCATCCTTGC-3′	
ELF3	F: 5′-GAGCAAAGAATACTGGGACTG-3′	165
	R: 5′-GAACGAAGAAACTTGAAAACG-3′	
IL-6	F: 5′- ATACCACCCACAACAGAC-3′	248
	R: 5′- GAACTCCAGAAGACCAGA-3′	
IL-10	F: 5′- GACCCACATGCTCCGAGA-3′	129
	R: 5′- CAACCCAAGTAACCCTTA-3′	
TNF-α	F: 5′- CTCCTCACCCACACCGTCA-3′	169
	R: 5′- GGTCCCCCTTCTCCAGC-3′	

**TABLE 2:** Real-time PCR reaction system.

Reagent	Dose /μL
2 × Real PCR EasyTM Mix-SYBR	10.0
Forward primer/(10 mol⋅ml^–1^)	0.8
Reverse primer/(10 mol⋅ml-1)	0.8
Template (cDNA/DNA)	1.0
50 × ROX reference	0.4
DNase-free ddH2O	7.0

### 2.9 Prediction of the *Salmonella* infection-related targets of Leo

To identify the potential *S. enteritidis* infection-related targets of Leo, a search for “leonurine” was performed in the PubChem database,^2^ and the obtained structure was optimized by Chem3D (ChemBio3D 14.0) and saved as an SDF file. The Pharm Mapper database^3^ and Swiss Target Prediction database^4^ were subsequently employed to predict the potential targets of Leo online, with the screened duplicates being merged into.xlsx files. Similarly, the potential targets of *Salmonella enteritidis* were retrieved from the Gene-Cards database^5^ using “*Salmonella enteritidis*” as the keyword, and the combined duplicates were removed through screening. Then, the online mapping tool platform system Venny 2.1^6^ was used to identify the shared Leo and *S. enteritidis* infection-related targets, which indicated the potential targets through which Leo combats *S. enteritidis* infection. The obtained shared targets were input into the STRING functional protein interaction network database^7^ with the species defined as “*Homo sapiens*,” and the protein–protein interaction (PPI) relationships were predicted under the screening condition of confidence ≥0.4, finally obtaining the PPI network depicting the molecular interactions of Leo with *S. enteritidis* and saving it as a TSV file. To further explore the relation-ships between the Leo and disease-related targets and understand the functions of the potential target genes identified through screening as well as relationships with various signaling pathways, the DAVID webtool^8^ was utilized to conduct Gene Ontology (GO) function and Kyoto Encyclopedia of Genes and Genomes (KEGG) pathway enrichment analyses of the potential targets related to Leo-mediated resistance to *S. enteritidis* infection. The species was set to “*Homo sapiens*,” and the core GO functions and pathways were sorted on the basis of the network node degree values.

### 2.10 Western blot analysis

The experimental groups included a control group, a Leo treatment group, an *S. enteritidis* challenge group, and a Leo treatment group, in which the concentration of Leo was set at 60 μmol/L and the *S. enteritidis* infection time was 6 h. Each group had three replicate groups. The four groups of cells were inoculated with *S. enteritidis* in 2% medium (98% DMEM with 2% fetal bovine serum) at an MOI of 1:100. Total protein was extracted from the cells, and the protein concentration was determined with a BCA kit. SDS–PAGE was carried out. The wet rotation method was used to complete the film rotation operation. Blocking was performed for 1 h with 3% BSA. The membranes were incubated with primary antibodies overnight at 4 °C on a shaker. The next day, the primary antibodies were collected and then washed five times with TBST, and the secondary antibodies were incubated and washed three times with TBST after incubation for 2 h at room temperature. Imaging was performed on a gel imager via an ultrasensitive ECL chemiluminescence kit, and ImageJ software was used to analyze the grayscale values of the protein bands. The relative protein expression of blank group was fixed, and the relative protein expression of other groups was observed, the technique was repeated 3 times. Antibodies against ZO-1, Occludin, and CD90 (Beijing Bioss Biotechnology Co., Ltd.) and EGFR, P65, CP, Lcn2, GPx4, LC3B, and GAPDH (Wuhan Sanying Biotechnology Co., Ltd.) were used.

## 3 Results

### 3.1 The inflammatory response of IEC-6 cells induced by *Salmonella enteritidis* was attenuated by Leo

**FIGURE 1:**
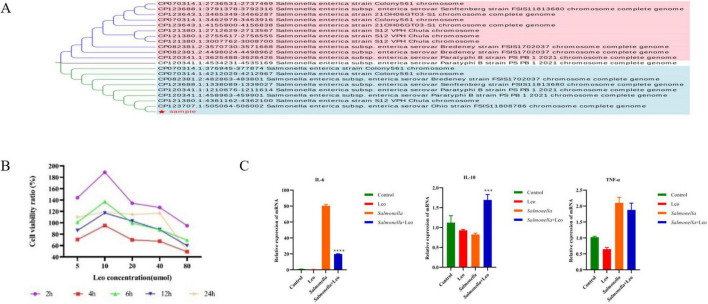
The inflammatory response of IEC-6 cells induced by *Salmonella enteritidis* was attenuated by Leo. **(A)** Phylogenetic tree analysis of *Salmonella enteritidis*; **(B)** effects of different times and doses of Leo on IEC-6 cell viability; **(C)** the expression of inflammatory factors IL-6, IL-10 and TNF-α changed. *n* = 3 per group. Two-tailed unpaired *t*-test; *p < 0.05, **p < 0.01, ***p < 0.001, ****p < 0.0001.

As shown in Figure 1A, nucleic acid sequence alignment of the bacterial liquid sequences revealed that the strain used for testing was indeed *S. enteritidis*. IEC-6 cells were treated with different concentrations (5, 10, 20, 40, or 80 μmol/L) of Leo for different durations (2, 4, 6, 12, or 24 h). As shown in Figure 1B, IEC-6 cells had the lowest viability at 4 h after Leo treatment, after which the cell viability increased with in-creasing administration time. Analysis revealed that low concentrations of Leo promoted cell growth and that high concentrations of Leo inhibited cell growth. Moreover, cell injury and apoptosis can produce an inflammatory response. To verify that Leo inhibits the cellular inflammatory response caused by *S. enteritidis*, RT–qPCR was used. Figure 1C shows that the mRNA expression of IL-6 in the control group and the Leo treatment group changed only slightly, but the mRNA expression of IL-6 in the *S. enteritidis* challenge group increased significantly. Compared with that in the *S. enteritidis* challenge group, the mRNA expression of IL-6 in the Leo treatment group was significantly lower. Additionally, IL-10 mRNA expression decreased, but not significantly, in the Leo treatment and *S. enteritidis* challenge groups compared with the control group, with the most significant decrease observed in the *S. enteritidis* group. Compared with that in the *S. enteritidis* challenge group, the mRNA expression of IL-10 in the Leo treatment group was significantly greater. The changes in TNF-α mRNA expression were consistent with the trend of IL-6 mRNA expression; that is, compared with the control group, the Leo treatment group presented little change, but the *S. enteritidis* challenge group presented a significant increase in the mRNA expression of TNF-α, whereas the Leo treatment group presented a significant decrease in the mRNA expression of TNF-α. The invasion of *S. enteritidis* into IEC-6 cells induced an inflammatory response, which was significantly inhibited by Leo treatment.

### 3.2 Leo inhibited the adhesion, invasion, and proliferation of *Salmonella enteritidis* in IEC-6 cells

**FIGURE 2:**
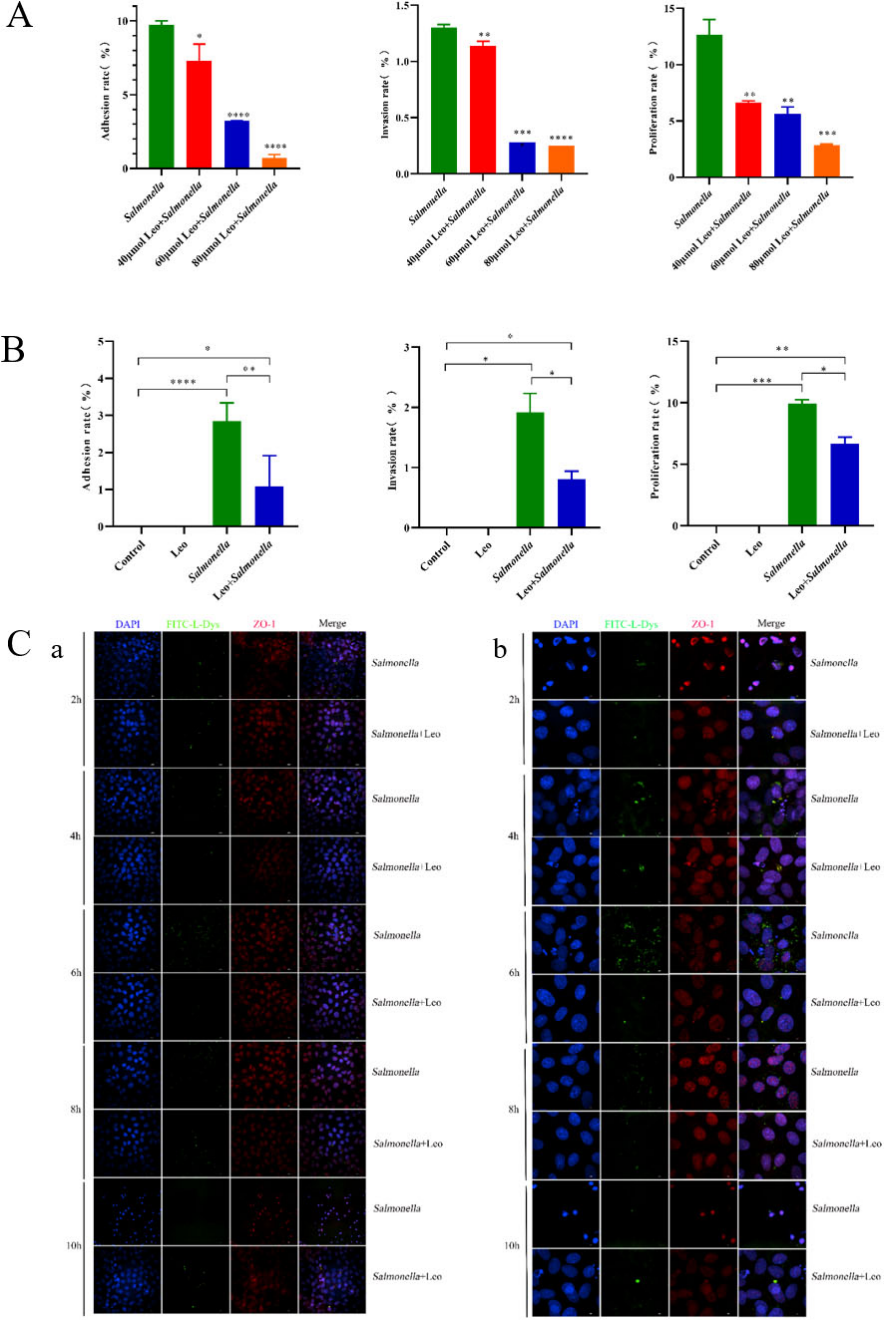
The compound Leo exhibits inhibitory effects on the adhesion, invasion, and proliferation of *Salmonella enteritidis* in IEC-6 cells. **(A)** Effects of different concentrations of Leo on adhesion, invasion and proliferation of *Salmonella* SPP; **(B)** effect of 60 μmol/L Leo on adhesion, invasion and proliferation of *Salmonella* SPP; **(C)** Immunofluorescence staining results. **C(a)** Immunofluorescence results are shown on the left. Scale bars: 10 μm. **C(b)** The right picture is a local magnification of Panel **C(a)**. Scale bars: 2 μm. *n* = 3 per group. Two-tailed unpaired *t*-test; *p < 0.05, **p < 0.01, ***p < 0.001, ****p < 0.0001.

To determine the optimal inhibitory concentration of Leo, IEC-6 cells were treated with different concentrations (40, 60, and 80 μmol/L) of Leo. As depicted in Figure 2A, Leo significantly inhibited the adhesion, invasion, and proliferation of *S. enteritidis*. Notably, the inhibitory effect was more pronounced at concentrations of 60 and 80 μmol/L. Considering the impact of Leo at 60 μmol/L on cell viability, this concentration was selected for subsequent experiments. As shown in Figure 2B, compared with treatment with *S. enteritidis* alone, treatment with Leo significantly decreased the adhesion rate, invasion rate, and proliferative rate of *S. enteritidis*. To further visualize the inhibitory effect of Leo on *S. enteritidis* invasion, the immunofluorescence results are presented in Figure 2C. DAPI was used for nuclear staining, ZO-1 was used to stain both the cell membrane and the cytoplasm; and FITC-L-Dys was used to label the peptidoglycan outer membrane of *S. enteritidis*. As shown in Figure 2C(a), *S. enteritidis* adhered to surrounding cells after a challenge period of 2 h, and the fluorescence intensity increased over time until reaching its peak at 6 h, after which the fluorescence intensity reduced as a result of the increased proliferation and replication of *S. enteritidis* progeny, which lacked green fluorescence. Figure 2C(b) shows that endocytosis facilitated *S. enteritidis* entry into cell membranes, where the green fluorescence of *Salmonella* interacted with the red fluorescent membrane dye, resulting in yellow fluorescence. Moreover, as shown in Figure 2C(b) (*Salmonella* + Leo group), after 6 h, *S. enteritidis* clearly induced changes in morphology within the nuclear membrane.

### 3.3 Administration of Leo mitigated the cellular damage induced by *Salmonella enteritidis*

**FIGURE 3:**
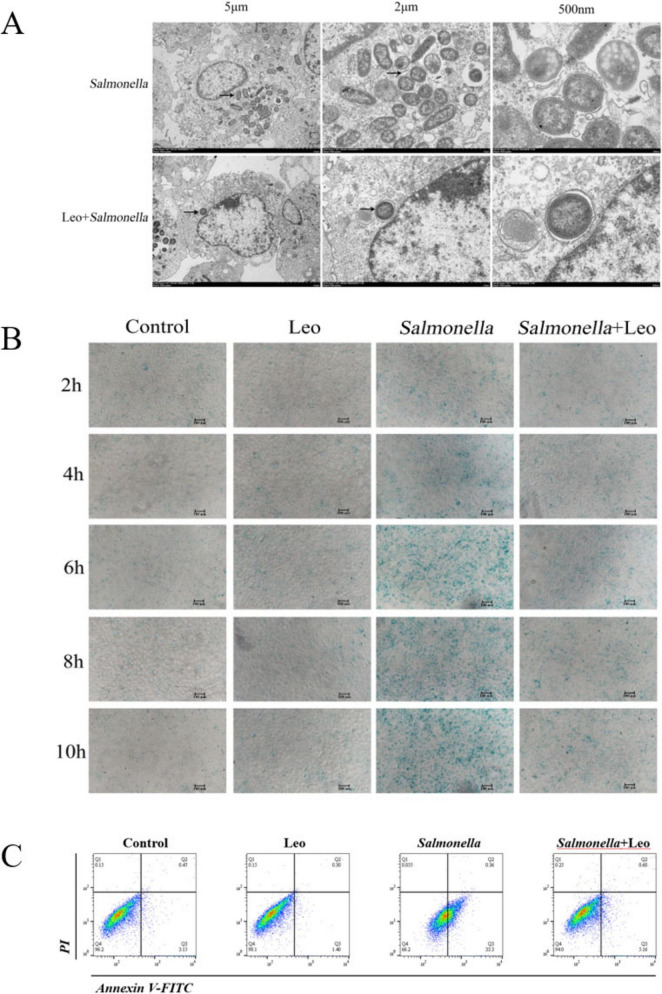
The administration of Leo could mitigate cellular damage induced by Salmonella enteritidis. **(A)** Transmission electron microscopy results. Scale bars: 5 μm, 2 μm and 500 nm; **(B)** results of β-galactosidase staining. Scale bars: 100 μm; **(C)** flow cytometry.

Since the most obvious inhibitory effect of Leo on the proliferation of *S. enteritidis* occurred at 6 h according to our previous immunofluorescence analyses, 6 h was selected as the time point for the observation of microscope *S. enteritidis* invasion via transmission electron microscopy, and the results are shown in Figure 3A. *S. enteritidis* vesicles (black arrows) in cells from both the *S. enteritidis* group and the Leo treatment group were between 500 nm and 2 μm in size and had a bilayer membrane structure. However, the number of *S. enteritidis* in the Leo treatment group was significantly lower than that in the *S. enteritidis* group, indicating that Leo could inhibit the intracellular proliferation of *S. enteritidis*. In addition, *S. enteritidis* caused nuclear membrane “invagination.” Since *S. enteritidis* can cause nuclear membrane invagination and induce cell apoptosis, the cells were further stained with β-galactosidase to study the protective effect of Leo on *S. enteritidis*-induced cell damage. As shown in Figure 3B, the degree of cell damage in the control group and the Leo treatment group was relatively small, and the cells could be metabolized by themselves, which was within the range of normal cell damage. The degree of cell damage increased with time in the *S. enteritidis* challenge group, whereas the degree of cell damage was significantly lower in the Leo treatment group than in the *S. enteritidis* challenge group, indicating that Leo could alleviate IEC-6 cell damage caused by *S. enteritidis*. Given the ability of *S. enteritidis* to cause cell damage, the effect of Leo on *S. enteritidis*-induced apoptosis was further investigated via flow cytometry. As shown in Figure 3C, the percentage of apoptotic cells in the control group was very low compared with that in the Leo treatment group, and the percentage of apoptotic cells in the Leo group was decreased, indicating that Leo could reduce the percentage of apoptotic normal IEC-6 cells. Compared with that in the *S. enteritidis* challenge group, the apoptosis rate was significantly lower in the Leo treatment group, indicating that Leo could indeed inhibit the apoptosis induced by *S. enteritidis*.

### 3.4 The molecular mechanism by which leonurine protects against *Salmonella enteritidis* infection was explored via network pharmacology

**FIGURE 4:**
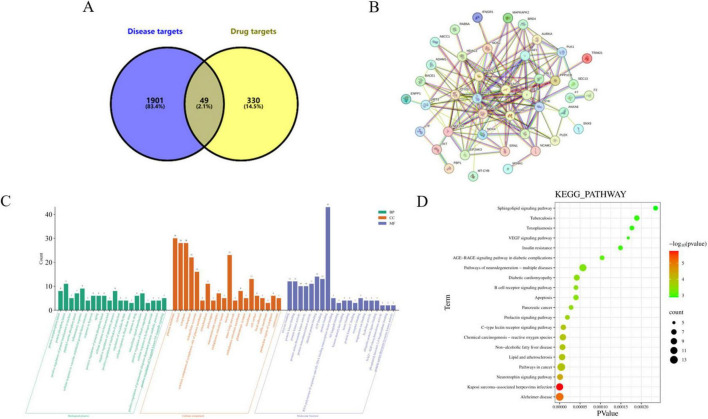
Network pharmacology was used to screen the interaction targets between leonurine and *Salmonella enteritidis*. **(A)** Venn diagram of intersection targets between Leo and *Salmonella enteritidis*; **(B)** protein interaction network of Leo and *Salmonella enteritidis* intersection targets; **(C)** GO function enrichment analysis bar chart; **(D)** KEGG signal pathway enrichment analysis bubble plot.

The structure of Leo was searched with “Leonurine” as the key word in the Pub-Chem database, and the targets of Leo were integrated into the Pharm Mapper data-base and Swiss Target Prediction database. A total of 379 drug targets were screened. The Gene Cards database was integrated with the keyword “*Salmonella enteritidis*,” and 1950 disease-related targets were obtained. The drug targets and disease-related targets were entered into the Venny 2.1 system, and 49 shared targets were obtained, as shown in Figure 4A. To further obtain the core targets, the 49 obtained shared tar-gets were imported into the STRING database, as shown in Figure 4B, and a PPI network with 48 nodes and 158 lines was obtained. The core targets were Akt serine/threonine kinase 1 (AKT1), signal transducer and activator of transcription 3 (STAT3), mammalian target of rapamycin (mTOR), glycogen synthase kinase 3 beta (GSK3B), V-Rel proto-oncogene, NF-kB RELA subunit, poly (ADP-ribose) polymerase 1 (PARP1), and prostaglandin endoperoxide synthase 2 (PTGS2). To analyze the biological processes involved in the anti-*S. enteritidis* infection effects of Leo, the data were imported into the DAVID webtool. With *P* < 0.05 as the screening condition, the top 20 data points were screened to generate a bar chart of the enriched GO terms, as shown in Figure 4C. A total of 99 biological process (BP) terms, which included protein phosphorylation, cell proliferation, negative regulation of apoptosis, proteolysis, the inflammatory response and other biological processes, were obtained. There were 23 enriched cellular component (CC) terms, including cytoplasm, mitochondria, cytoplasm, endosomes, vesicles, etc. Finally there were 17 enriched molecular function (MF) terms, including enzyme binding, NF-κB binding, ATP binding, serine/threonine protein kinase activity, protein deacetylase activity, G-protein coupled acetylcholine receptor activity, etc. To analyze the signaling pathways regulated by Leo in the context of *S. enteritidis* infection, the data were imported into DAVID, and 102 enriched KEGG pathways were obtained via KEGG pathway enrichment analysis. With *P* < 0.05 as the screening criterion, 93 enriched KEGG pathways were obtained. The top 20 pathways are visualized in a bubble diagram, as shown in Figure 4D. The results revealed that the pathways involved in the resistance of Leo to *S. enteritidis* infection included cell apoptosis, the VEGF signaling pathway, the insulin signaling pathway, and pathways related to cancer and autophagy. Among these pathways, the Fc epsilon RI signaling pathway, Fcγ-R-mediated phagocytosis, the T-cell receptor signaling pathway, the MAPK signaling pathway, and the mTOR signaling pathway are directly related to the inflammatory response.

### 3.5 Transcriptome profile of IEC-6 cells infected with *Salmonella enteritidis*

**FIGURE 5:**
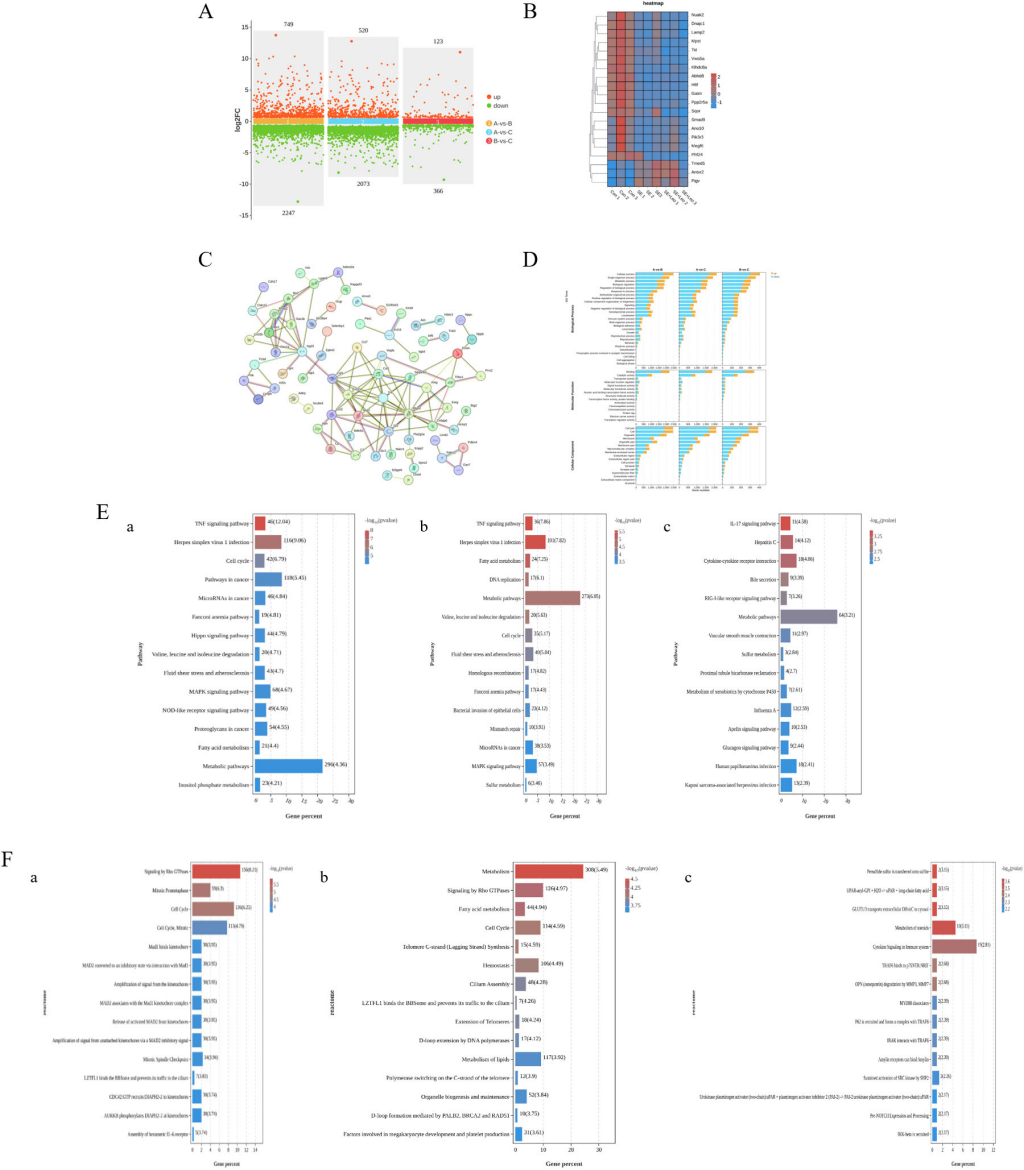
Transcriptome profile of IEC-6 cells infected with *Salmonella enteritidis*. **(A)** Statistical map of differential genes; **(B)** heat map of differential gene distribution; **(C)** PPI map of core targets of the transcriptome; **(D)** GO enrichment analysis of differentially expressed genes among the three groups; **(E)** KEGG enrichment analysis of differentially expressed genes among the three groups. **(a)** KEGG enrichment analysis of blank group and *Salmonella* challenge group. **(b)** KEGG enrichment analysis between the blank group and the Leo treatment group. **(c)** KEGG enrichment analysis between Leo treatment group and *Salmonella* challenge group; **(F)** Reactome enrichment analysis of differentially expressed genes among the three groups. **F(a)** Reactome enrichment analysis of blank group and *Salmonella* challenge group. **F(b)** Reactome enrichment analysis between the blank group and the Leo treatment group. **F(c)** Reactome enrichment analysis between Leo treatment group and *Salmonella* challenge group. *n* = 3 per group.

A total of 23091 genes were analyzed, and 14620 genes were detected, including 12846 genes in control group A1, 13008 genes in control group A2, 12844 genes in control group A3, and 12671 genes in *S. enteritidis* challenge group B1. The *S. enteritidis* challenge group B2 contained 12564 genes, the *S. enteritidis* challenge group B3 contained 12652 genes, the Leo treatment group C1 contained 12583 genes, the Leo treatment group C2 contained 12538 genes, and the Leo treatment group C3 contained 12404 genes; is the results are shown in Supplementary Table 1. The gene abundance in each group was visualized and analyzed. According to Supplementary Figure 1, the nonabnormal data were scattered, and the kernel density distribution was concentrated, indicating that the quality of the samples was sufficient and that they could be used for subsequent analysis. The coordinates of PC1 represent the first principal component, and the percentages in parentheses reveal the specific contribution of the first principal component to the sample variance. The coordinates of PC2 represent the second principal component, and the percentages in parentheses reveal the extent to which the second principal component contributes to the sample variance. The colored points in the figure indicate individual samples separately. As shown in the principal component analysis (PCA) diagram in Supplementary Figure 2, control groups A1, A2, and A3 were in the first quadrant; *S. enteritidis* challenge groups B1, B2, and B3 were in the third and fourth quadrants; and Leo treatment groups C1, C2, and C3 were in the second quadrant, with high intragroup homogeneity. As shown in Figure 5A, compared with those in the control group, 749 genes were upregulated and 2247 genes were downregulated in the *S. enteritidis* challenge group, and 520 genes were upregulated and 2073 genes were downregulated in the Leo treatment group. Compared with those in the *S. enteritidis* challenge group, 123 genes were upregulated, and 366 genes were downregulated in the Leo treatment group. The genes whose expression levels significantly changed between the different groups are shown in Figure 5B. The predicted targets identified through network pharmacology were compared with the results obtained from transcriptome sequencing analysis (Supplementary Figure 3). The shared targets between the two target lists were NOS2, PTGS2 and mTOR. There were 128 core targets in the transcriptome, and 128 targets were used to construct a PPI network diagram. Among them, PTGS2, CXCL2, TNF, NOS2, LCN2, CSF2, etc., were the genes with high *p*-values, as shown in Figure 5C.

Gene Ontology enrichment analysis was performed on the differentially expressed genes among the three groups, and the enrichment results are shown in Figure 5D. The enriched BP terms included cellular process, metabolic process, biological regulation, vesicle transport, stimulus response, positive regulation of biological process, negative regulation of biological process, immune system process, and synaptic transmission. The enriched MF terms included binding, catalytic activity, transporter activity, molecular sensor activity, nucleic acid transcription factor activity, structural molecule activity, antioxidant activity, chemical adsorption activity, chemotactic activity, etc. The enriched CC terms included cells, cell membranes, organelle membranes, synaptic parts, the extracellular matrix, and nucleoids.

Kyoto Encyclopedia of Genes and Genomes enrichment analysis was performed on the differentially expressed genes among the three groups, and the top 15 enriched pathways were visualized. As shown in Figure 5E, compared with those in the control group, the genes expressed in the *S. enteritidis* challenge group were significantly associated with the TNF signaling pathway, autophagy, the cell cycle, the cancer pathway, the MAPK signaling pathway, the NOD-like receptor signaling pathway, focal adhesion, bacterial invasion of epithelial cells, the Rap1 signaling pathway, the IL-17 signaling pathway, cytokine–cytokine receptor interaction and endocytosis JAK–STAT signaling pathway, the Toll–like receptor signaling pathway, the FoxO signaling pathway, the mTOR signaling pathway, *Salmonella* infection, the PPAR signaling pathway, etc., [Figure 5E(a)]. The genes expressed in the Leo treatment group were associated with the TNF signaling pathway; fatty acid metabolism; DNA replication; the metabolic pathway; valine, leucine and isoleucine degradation; the cell cycle; the MAPK signaling pathway; endocytosis; the IL-17 signaling pathway; the PPAR signaling pathway; the P53 signaling path-way; and *Salmonella* infection [Figure 5E(b)]. Compared with those in the *Salmonella* challenge group, the genes in the Leo treatment group were associated with the IL-17 signaling pathway, hepatitis C, cytokine–cytokine receptor interaction, bile secretion, the cAMP signaling pathway, the Toll–like receptor signaling pathway, the PI3K–Akt signaling pathway, etc., [Figure 5E(c)].

Reactome enrichment analysis was performed on the differentially expressed genes among the three groups, and the top 15 enriched terms were visualized. As shown in Figure 5F, compared with those in the control group, the genes expressed in the *S. enteritidis* challenge group were significantly associated with premitotic metaphase, the cell cycle, mitosis, Mad1 binding to the centromere, MAD2 conversion to a repressive state by interacting with Mad1, tyrosine phosphorylation of STAT1 and STAT3 by the IL6 receptor, kinesin, etc., [Figure 5F(a)]. The genes expressed in the Leo treatment group were associated with metabolism, fatty acid metabolism, the cell cycle, telomere c chain synthesis, ciliary assembly, telomere elongation, DNA polymerase-mediated d loop extension, lipid metabolism, etc., [Figure 5F(b)]. Compared with those in the *S. enteritidis* challenge group, the genes in the Leo treatment group were associated with the transfer of persulfide sulfur to sulfite, steroid metabolism, cytokine signaling in the immune system, osteopontin degradation through MMP3 and MMP7, p62 recruitment and formation of a complex with TRAF6, IRAK interaction with TRAF6, SHP2 continuous activation of SRC kinase, phosphorous of PD-1 acidification, CD28-dependent PI3K/Akt signaling pathway, etc., [Figure 5F(c)].

### 3.6 The expression of differentially expressed genes and proteins was assessed via RT–qPCR and Western blot analysis

**FIGURE 6:**
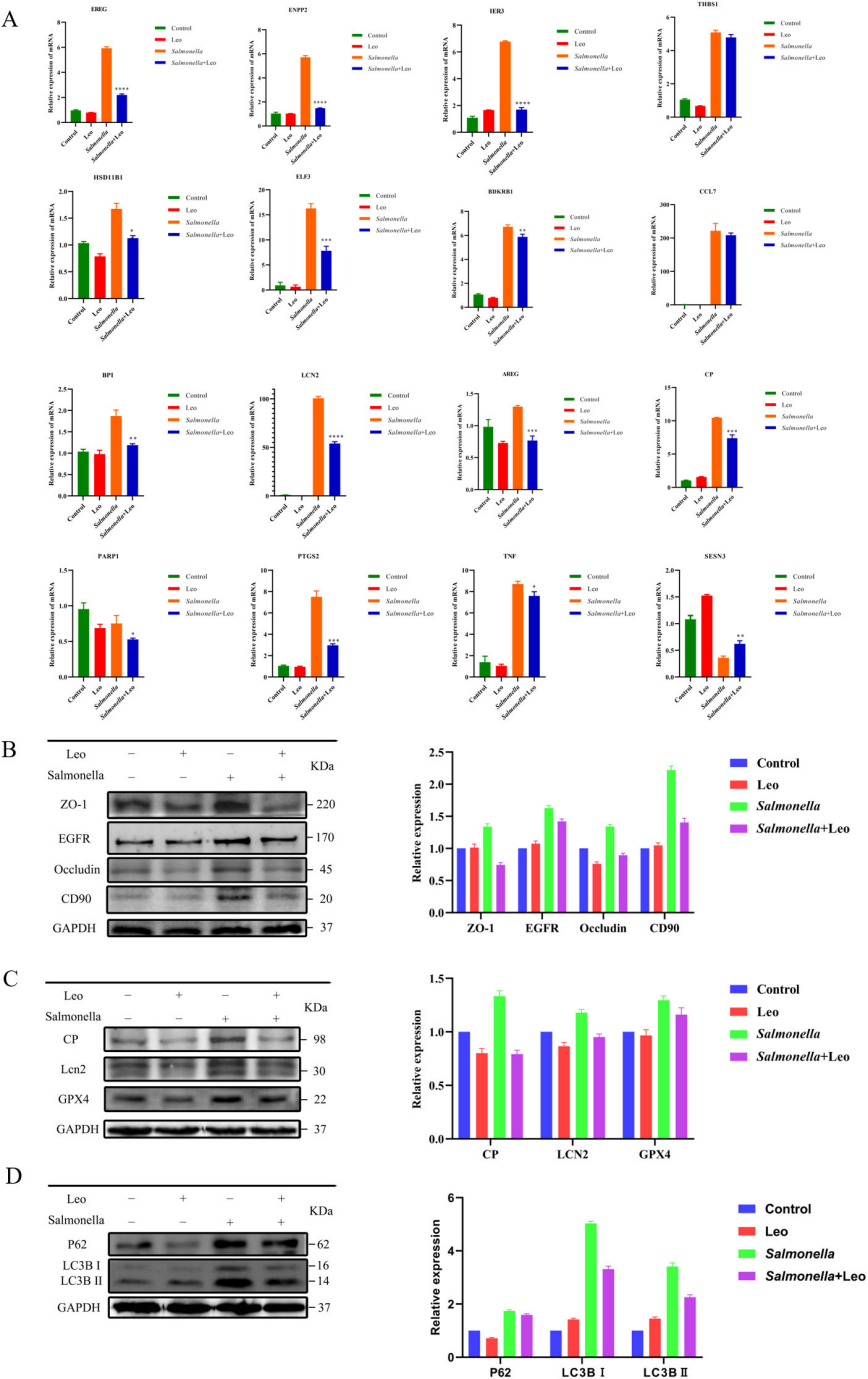
The expression of differential genes and proteins was assessed using RT-qPCR and Western blot techniques. **(A)** Changes in mRNA content of core targets in each group; **(B)** Western Blot was used to detect the expression of membrane proteins; **(C)** The expression of ferroptosis protein was detected by Western Blot; **(D)** Western Blot was used to detect the expression of autophagy proteins. *n* = 3 per group. Two-tailed unpaired *t*-test; *p < 0.05, **p < 0.01, ***p < 0.001, ****p < 0.0001.

To screen the core targets and verify the accuracy of the transcriptomic analysis, RT–qPCR was used to measure the mRNA expression of the differentially expressed genes. The results are shown in Figure 6A. Compared with those in the control group, BPI (bactericidal permeability increasing protein), HSD11B1 (11-β-hydroxysteroid de-hydrogenase 1), IER3 (early response gene 3), LCN2 (lipocalin 2), TNF (tumor necrosis factor), PTGS2 (prostaglandin peroxidase 2), THBS1 (lipocalin 2) Thromtomodulin type I), AREG (pancreatin-like growth factor), BDKRB1 (angiotensin-like peptide B1 receptor), CCL7 (chemokine ligand 7), CP (ceruloplasmin), ELF3 (E74 like factor 3), ENPP2 (alkaline phosphatase 2), and EREG (epithelial growth factor) were upregulated in the *S. enteritidis* challenge group. Compared with that in the *S. enteritidis* challenge group, the expression of these genes was lower in the Leo treatment group, and the RT–qPCR results were consistent with the transcriptome sequencing results. Compared with that in the control group, the expression of SESN3 was significantly lower in the *S. enteritidis* challenge group, and the expression of Sesn3 was significantly greater in the Leo treatment group than in the *S. enteritidis* challenge group. The RT–qPCR results were consistent with the transcriptome sequencing results.

*Salmonella enteritidis* invades cells through their cell membranes, and the early stages of infection involve vesicle transport and other biological processes. Therefore, measuring the expression of membrane proteins is helpful for understanding the anti-*S. enteritidis* infection effects of Leo. T As shown in Figure 6B, compared with those in the control group, the protein expression levels of ZO-1, EGFR, Occludin and CD90 in the *S. enteritidis* challenge group were significantly increased. Compared with those in the *S. enteritidis* challenge group, the protein expression levels of ZO-1, EGFR, Occludin and CD90 in the Leo treatment group were significantly lower. These results indicated that *S. enteritidis* invasion could increase the expression of membrane proteins and that Leo treatment could significantly reduce the expression of membrane proteins. RT–qPCR experiments verified that Leo inhibition of *S. enteritidis* caused changes in Lcn2 and CP contents, which play key roles in the ferroptosis signaling pathway; these findings verified that Leo inhibition of *S. enteritidis* invasion inhibited the ferroptosis signaling pathway. As shown in Figure 6C, the protein expression levels of CP, Lcn2 and GPX4 in the *S. enteritidis* challenge group were significantly greater than those in the control group. In the Leo treatment group, the protein expression of CP, Lcn2, and GPX4 was significantly lower than that in the *S. enteritidis* challenge group. These results indicate that Leo inhibited the activation of the ferroptosis signaling pathway caused by *S. enteritidis* invasion. Previous network pharmacology and RNA-seq studies verified that Leo could be involved in the regulation of the autophagy signaling pathway. Therefore, the expression of the autophagy markers P62 and LC3B was detected. The results of the Western blot analysis are shown in Figure 6D. The protein expression of P62, LC3BI and LC3BII in the *S. enteritidis* challenge group was significantly increased. Compared with those in the *S. enteritidis* challenge group, the protein expression levels of P62, LC3BI and LC3BII in the Leo treatment group were significantly lower. These results indicated that *S. enteritidis* activates the autophagy response and that Leo suppressed the autophagy response.

## 4 Discussion

*Salmonella*, a pathogen that is transmitted primarily through the consumption of contaminated meat and meat products, has caused significant morbidity and mortality worldwide. The increase in antibiotic resistance has emerged as a major public health threat in the 21st century. Consequently, there is an urgent need to develop alternatives to conventional antibiotics or identify effective therapeutic strategies to address the challenge of antibiotic resistance in *Salmonella* infection. *Salmonella* has evolved multiple strategies to invade host cells and establish systemic infections. There are two critical steps for its successful infection: membrane ruffling and internalization. Mechanistic studies have revealed that *Salmonella enterica* initially adheres to host cells through surface adhesion factors, which bind to specific receptors on the host cell surface, thereby facilitating the preliminary attachment phase (PLoS One Staff, 2024; Drolia et al., 2018; Sutton et al., 2023). Our study demonstrated that Leo, at a concentration of 60 μmol/L, exhibited no observable cytotoxicity or antimicrobial activity, indicating that this concentration had no adverse effects on cellular or bacterial growth. Consequently, this concentration was selected to evaluate the impact of Leo on bacterial adhesion, invasion, and intracellular survival. Notably, colony counting assays revealed that Leo significantly inhibited *Salmonella* adhesion, invasion, and intracellular proliferation. Preventing *Salmonella* adhesion is a critical strategy in disrupting bacterial pathogenesis and colonization during early infection. Intriguingly, minimal bacterial adhesion or invasion was detected on the cell surface within the first 2 h post infection, with peak levels observed at 6 h, followed by a gradual decline. This phenomenon may be attributed to the initial increase in the number of *Salmonella* progeny over time, combined with the absence of green fluorescence in offspring bacteria after 6 h due to methodological limitations in staining. Strikingly, at 10 h postinfection, the nuclei in the *Salmonella* infected cells exhibited pronounced fragmentation, whereas the nuclei in the Leo-treated cells remained intact. Additionally, flow cytometry and cytopathic effect analyses revealed a markedly increased apoptosis rate in the *Salmonella* infected group, which was significantly reduced by Leo treatment. These findings collectively corroborate that Leo effectively mitigates the detrimental effects of *Salmonella* on host cells.

Following adhesion to intestinal epithelial cells, *Salmonella* facilitates its invasion by activating and injecting a repertoire of effector proteins. These virulence factors modulate host cell gene expression and cytoskeletal organization, disrupt tight junction structures, and subsequently promote bacterial internalization, triggering inflammatory responses and apoptosis. Maintaining intestinal barrier integrity has been shown to block *Salmonella* entry into epithelial cells, thereby mitigating bacterium induced damage in both *in vitro* and *in vivo* intestinal models. Accumulating evidence underscores the critical role of tight junction proteins, such as ZO-1 and Occludin, in preserving cellular polarity and establishing the paracellular barrier that regulates selective permeability to solutes and ions (Anderson and Van Itallie, 2009; Rao et al., 2016). Studies have demonstrated that *Salmonella* increases epithelial permeability by altering the expression of tight junction proteins, including ZO-1 and Occludin, thereby facilitating bacterial translocation via the paracellular pathway, regardless of invasive-ness. Invasive bacteria further activate cytoskeletal remodeling mechanisms, promoting the synthesis and aggregation of cytoskeletal proteins. This process aids in bacterial internalization while restricting bacterial motility, potentially enhancing bacterial digestion or expulsion. Upon contact with host cells, *Salmonella* induces membrane ruffling, enabling phagocytosis and the formation of double-membrane-bound vesicles (*Salmonella* -containing vacuoles) within intestinal epithelial cells. Consistent with these findings, our study revealed that *Salmonella* infection significantly upregulated the expression of ZO-1 and Occludin at both the protein and mRNA levels. This upregulation likely promotes tight junction redistribution and reorganization, facilitating the invasion of IEC-6 cells (an intestinal epithelial cell line). Notably, Leo pre-treatment counteracted this process by modulating membrane protein expression, thereby blocking *Salmonella* adhesion and invasion. Transmission electron microscopy further confirmed that *Salmonella* rearranges the cytoskeleton to enter, survive, and proliferate within host cells. Importantly, Leo treatment markedly suppressed intracellular bacterial replication. Intriguingly, while prior studies confirmed that Leo lacks direct bactericidal activity against *Salmonella*, we hypothesize that its anti-infection effects are mediated through enhancing host immune recognition, thereby bolstering barrier defense mechanisms against pathogen invasion.

To further dissect the mechanism by which Leo combats *Salmonella* infection, this study employed an integrated network pharmacology and transcriptomics approach, identifying 129 key targets predominantly involved in ferroptosis, apoptosis, inflammation, autophagy, MAPK signaling, and mTOR signaling. Intestinal epithelial cells (IECs) play a pivotal role in orchestrating adaptive immune responses against pathogen invasion, which are primarily mediated by the production of cytokines such as interleukins (ILs) and tumor necrosis factor-alpha (TNF-α). These signaling molecules regulate immune cell recruitment, barrier repair, and pathogen clearance (Bahrami et al., 2011; Beck et al., 2016; Devriendt et al., 2010; Kagnoff and Eckmann, 1997; Carey and Kostrzynska, 2013; Turner et al., 2019; Luo and Zheng, 2016). Numerous studies have shown that *Salmonella* invasion of epithelial cells triggers a proinflammatory cytokine storm (e.g., upregulated IL-6, TNF-α) alongside the suppression of immunoregulatory cytokines (e.g., downregulated IL-10), exacerbating tissue damage and barrier dysfunction. Strikingly, our findings demonstrate that Leo pretreatment effectively ameliorates *Salmonella* driven inflammatory responses in intestinal epithelial cells (IECs) by restoring normal cytokine levels. Mechanistically, Leo counteracts the pathogen-induced imbalance, suppressing hyperactivation of proinflammatory mediators while increasing IL-10 expression, thereby mitigating excessive immune activation and promoting tissue repair. Consistent with prior findings by Zheng et al. (2021) demonstrating the efficacy of Leo in alleviating DSS-induced colitis through intestinal mucosal repair and the suppression of proinflammatory cytokine release, this study further revealed its pivotal role in preventing *Salmonella* infection via iron metabolism modulation. During *Salmonella* invasion, host phagocytes and pathogens compete strongly for iron, a critical nutrient for bacterial survival. Leo disrupts this battle by enhancing host iron sequestration, a strategy synergized by its up-regulation of the antimicrobial peptide lipocalin-2 (LCN2). LCN2 directly binds bacterial siderophores (e.g., enterobactin), starving *Salmonella* of iron and impeding its proliferation. This dual action–repairing barrier integrity and enforcing nutritional immunity–positions Leo as a multifaceted therapeutic agent against enteric pathogens (Golonka et al., 2019). LCN2, identified as a core therapeutic target through integrated transcriptomic and network pharmacology screening, plays a pivotal role in *Salmonella* infection. Upon pathogen challenge, *Salmonella* triggers the host’s nutritional immune system to upregulate LCN2 expression, which promotes the binding of LCN2 to enterobactin a catecholate-type siderophore predominantly synthesized by *Salmonella* Typhimurium and *E. coli*–thereby starving the pathogen of iron. Paradoxically, proinflammatory cytokines (e.g., IL-1β) have been shown to decrease LCN2 expression. Intriguingly, Leo treatment reduces LCN2 levels through two mechanisms, namely, anti-inflammatory action, in which Leo suppresses the expression of PTGS2 (COX-2) and IL-1β, indirectly downregulating LCN2 by mitigating cytokine-driven inhibition, and pathogen load control. By limiting *Salmonella* invasion, Leo reduces the bacterial burden, consequently diminishing the host’s demand for LCN2-mediated iron sequestration. This bidirectional regulation of LCN2 highlights the ability of Leo to fine-tune host–pathogen interactions–bolstering iron restriction during infection while preventing excessive LCN2 depletion under inflammatory stress (Flo et al., 2004; Hamm and Yuste, 2016; Sadeghmousavi et al., 2020). Moreover, Lcn2 and GPX4 are markers of ferroptosis in cells (Dixon et al., 2012; Shimada et al., 2016; Wang et al., 2020), and research has shown that *Salmonella* can inhibit the expression of GPX4 and induce ferroptosis in cells (Chen et al., 2023). Copper blue protein (CP) is a copper containing α 2-globulin that can oxidize ferrous ions into iron ions, promote the loss of intra-cellular iron to the extracellular environment, and inhibit iron death (Sun et al., 2024). This study also demonstrated that *Salmonella* can activate the ferroptosis signaling pathway after entering host cells (Gao et al., 2023; Zheng et al., 2022). Leo can participate in the ferroptosis signaling pathway and inhibit the intracellular proliferation of *Salmonella* by regulating the expression of Lcn2, GPX4, and CP. MTOR is a serine/threonine kinase that mainly participates in processes such as cell growth, apoptosis, and autophagy (Saxton and Sabatini, 2017). Research has confirmed that *Salmonella* has evolved a complex escape mechanism that can activate mTOR and suppress immune responses to inhibit autophagy or evade pathogen recognition and capture during autophagy (Kohler and Roy, 2017). Our results demonstrated that Leo treatment in *Salmonella* -infected cells significantly increased the LC3-II/LC3-I ratio and downregulated p62 expression, indicating increased autophagic activity and flux to suppress intracellular bacterial proliferation. These findings collectively suggest that Leo is a promising and effective antibacterial agent for combating *Salmonella* infection.

Overall, Leo significantly inhibits *Salmonella* induced cellular apoptosis and alleviates cellular damage by increasing IL-10 mRNA expression while suppressing the mRNA expression of proinflammatory mediators, including IL-1β, COX-2, and TNF-α. Additionally, Leo inhibits bacterial invasion by modulating membrane protein expression. Furthermore, it restricts intracellular *Salmonella* proliferation through dual regulation of the ferroptosis and autophagy signaling pathways. As a lead compound, Leo exhibits promising anti *Salmonella* activity without exerting selective pressure on bacterial growth, a critical advantage in addressing widespread antibiotic resistance. Nevertheless, further studies are warranted to identify the specific target proteins mediating the antibacterial effects of Leo.

## Data Availability

The original contributions presented in the study are publicly available. This data can be found here: NCBI BioProject, accession PRJNA1433358.

## Footnotes

1
https://www.ncbi.nlm.nih.gov/

2
https://pubchem.ncbi.nlm.nih.gov/

3
http://lilab-ecust.cn/pharmmapper/index.html

4
http://swisstargetprediction.ch/

5
https://www.genecards.org/

6
https://bioinfogp.cnb.csic.es/tools/venny/index.html

7
https://cn.string-db.org/

8
https://davidbioinformatics.nih.gov/
