# Supplementary material for: Transcriptomics and network pharmacology reveal the molecular mechanisms through which leonurine protects against Salmonella enteritidis infection in IEC-6 cells
Source: Front Microbiol. 2026 Apr 10;16:1665245. doi: 10.3389/fmicb.2025.1665245 (PMC13106393; doi:10.3389/fmicb.2025.1665245)
Supplement: Supplementary file 1 [file Data_Sheet_1.docx]

Supplementary Material

# Supplementary Figures and Tables

**Supplementary Table 1** Number of genes in three groups of samples

| Sample | Total_Genes | sequenced_Total_Genes(%) |
| --- | --- | --- |
| all | 23091 | 14620 (63.31%) |
| A1 | 23091 | 12846 (55.63%) |
| A2 | 23091 | 13008 (56.33%) |
| A3 | 23091 | 12844 (55.62%) |
| B1 | 23091 | 12671 (54.87%) |
| B2 | 23091 | 12564 (54.41%) |
| B3 | 23091 | 12652 (54.79%) |
| C1 | 23091 | 12583 (54.49%) |
| C2 | 23091 | 12538 (54.30%) |
| C3 | 23091 | 12404 (53.72%) |

# Supplementary Figures


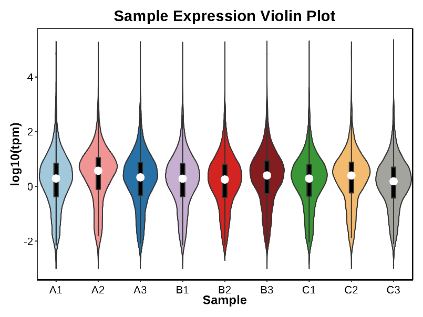


**Supplementary Figure 1.** Transcriptome violin diagram


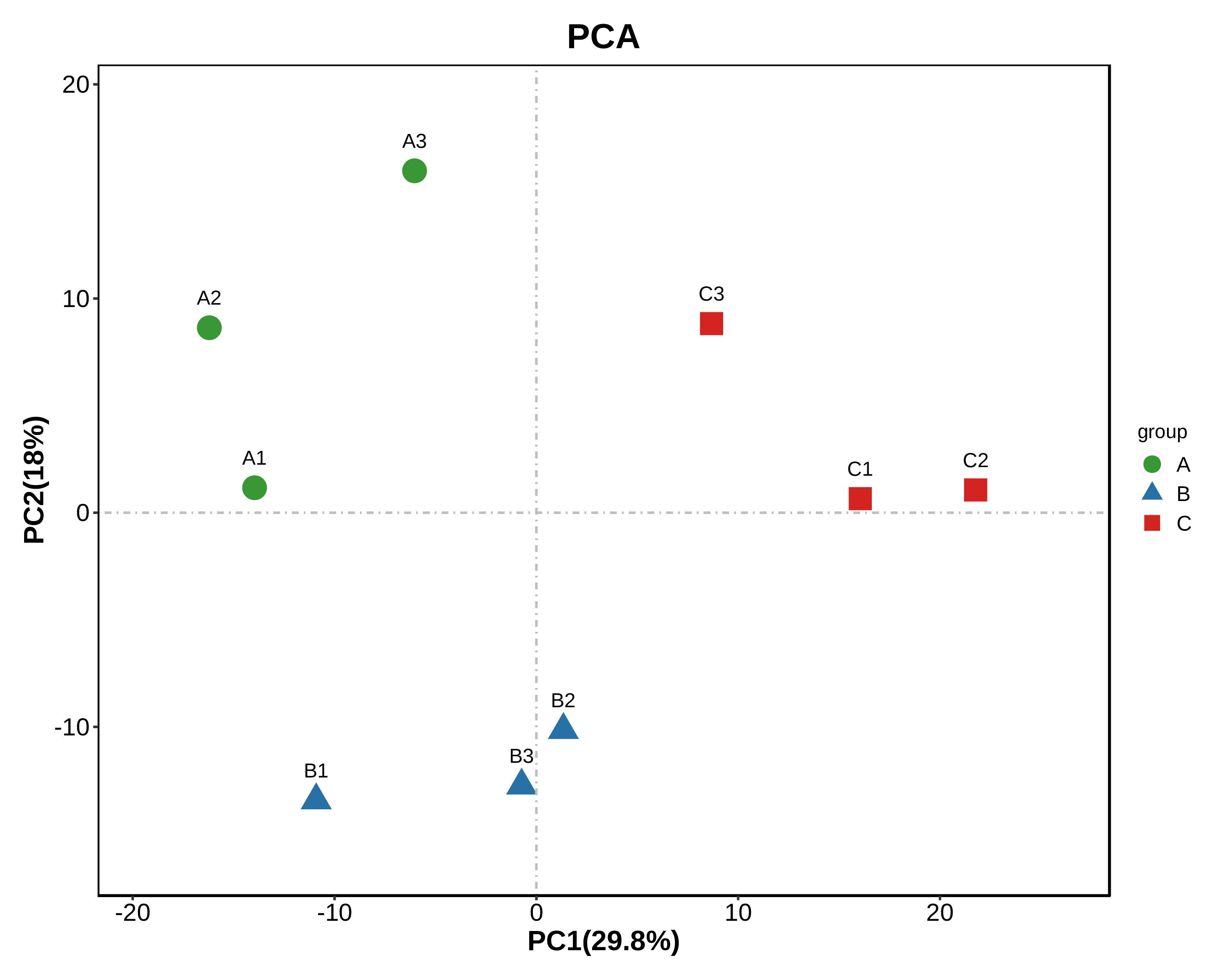


**Supplementary Figure 2.** Transcriptome PCA map


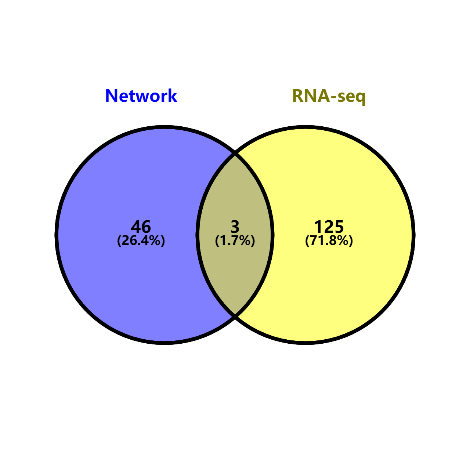


**Supplementary Figure 3.** Venny map of the intersection target of transcriptome and network pharmacology
